# Preparation, Characterization, and Radiolabeling of [^68^Ga]Ga-NODAGA-Pamidronic Acid: A Potential PET Bone Imaging Agent

**DOI:** 10.3390/molecules25112668

**Published:** 2020-06-09

**Authors:** Zarif Ashhar, Nor Azah Yusof, Fathinul Fikri Ahmad Saad, Siti Mariam Mohd Nor, Faruq Mohammad, Wan Hamirul Bahrin Wan Kamal, Muhammad Hishar Hassan, Hazlina Ahmad Hassali, Hamad A. Al-Lohedan

**Affiliations:** 1 Chemistry Department, Faculty of Science, Putra Malaysia University, Selangor, Serdang 43400, Malaysia; zarifnaim@gmail.com (Z.A.); smariam@upm.edu.my (S.M.M.N.); 2 Pharmacy Department, National Cancer Institute, Putrajaya 62250, Malaysia; 3 Centre for Diagnostic Nuclear Imaging (CDNI), Faculty of Medicine and Health Sciences, Putra Malaysia University, Selangor, Serdang 43400, Malaysia; fathinulfikri@upm.edu.my (F.F.A.S.); hishar.hassan@gmail.com (M.H.H.); 4 Surfactants Research Chair, Chemistry, College of Science, King Saud University, Riyadh 11451, Saudi Arabia; hlohedan@ksu.edu.sa; 5 Bahagian Teknologi Perubatan, Malaysia Nuclear Agency, Selangor, Kajang 43600, Malaysia; mirul@nuclearmalaysia.gov.my (W.H.B.W.K.); hazlinaahmad@nuclearmalaysia.gov.my (H.A.H.)

**Keywords:** [^68^Ga]Ga-NODAGA-pamidronic acid, radiopharmaceuticals, positron emitting tomography (PET), contrasting agents, bone metastases, ion suppression, bisphosphonate

## Abstract

Early diagnosis of bone metastases is crucial to prevent skeletal-related events, and for that, the non-invasive techniques to diagnose bone metastases that make use of image-guided radiopharmaceuticals are being employed as an alternative to traditional biopsies. Hence, in the present work, we tested the efficacy of a gallium-68 (^68^Ga)-based compound as a radiopharmaceutical agent towards the bone imaging in positron emitting tomography (PET). For that, we prepared, thoroughly characterized, and radiolabeled [^68^Ga]Ga-NODAGA-pamidronic acid radiopharmaceutical, a ^68^Ga precursor for PET bone cancer imaging applications. The preparation of NODAGA-pamidronic acid was performed via the *N*-Hydroxysuccinimide (NHS) ester strategy and was characterized using liquid chromatography-mass spectrometry (LC-MS) and tandem mass spectrometry (MS^n^). The unreacted NODAGA chelator was separated using the ion-suppression reverse phase-high performance liquid chromatography (RP-HPLC) method, and the freeze-dried NODAGA-pamidronic acid was radiolabeled with ^68^Ga. The radiolabeling condition was found to be most optimum at a pH ranging from 4 to 4.5 and a temperature of above 60 °C. From previous work, we found that the pamidronic acid itself has a good bone binding affinity. Moreover, from the analysis of the results, the ionic structure of radiolabeled [^68^Ga]Ga-NODAGA-pamidronic acid has the ability to improve the blood clearance and may exert good renal excretion, enhance the bone-to-background ratio, and consequently the final image quality. This was reflected by both the in vitro bone binding assay and in vivo animal biodistribution presented in this research.

## 1. Introduction

Cancer is one of the leading causes of mortality worldwide. The efforts in implementing an accurate diagnosis, particularly in cancer staging, are necessary for optimal patient management. Approximately 18.1 million new cancer cases were reported in 2018 [1], and it is foreseen to rise just by growth and ageing of the population. In Malaysia, bone cancer is the third most prevalent cancer in males, with an age group of 15–24 years old [2]. Early diagnosis of bone cancer is essential to prevent any skeletal-related events, and for that, the use of cost-effective single-photon emission computed tomography (SPECT) imaging guided by [^99m^Tc]Tc-medronic acid ([^99m^Tc]Tc-MDP) is suggested for staging metastatic high-risk prostate cancer and breast cancer with adverse prognostic factors [3].

The latest survey in Malaysia discovered that the usage of [^99m^Tc]Tc-MDP was the highest amongst other SPECT radiopharmaceuticals [4]. However, SPECT imaging has several limitations, primarily in quantifying the treatment response [5]. Positron emission tomography (PET) imaging has appeared as an essential imaging tool for providing an accurate cancer diagnosis, staging, and restaging [6]. Since the introduction of the ^68^Ge/^68^Ga generator in early 2000, interests in developing gallium-68 (^68^Ga) radiopharmaceuticals have been rampant due to its physical properties (positron emission, t_1/2_ = 68 min). Besides, the design of a ^68^Ga-radiopharmaceutical has contributed to personalized medicine through its lutetium-177 twin for therapy. Hence, the use of radiopharmaceuticals has been proven to improve therapy selection, predict adverse effects, and also monitor therapy response [7].

**Figure 1:**
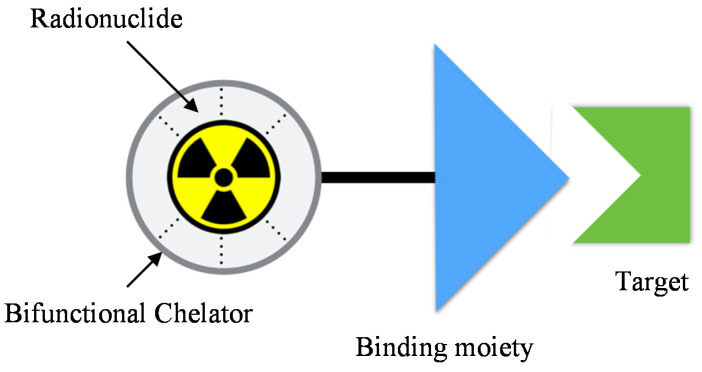
The design of a target mediated radiopharmaceutical consists of a radionuclide complexed to a bifunctional chelator via a process named radiolabeling. The bifunctional chelator is covalently conjugated to a binding moiety, which acts as a vehicle for the radiopharmaceutical to bind to a specific target.

Recent developments, in particular to ^68^Ga-labeled bisphosphonates as a potential for PET bone metastases imaging, have been studied [8]. Unlike [^99m^Tc]Tc-MDP, most of the ^68^Ga-labeled bisphosphonates studied were indirectly chelated to the binding moiety [9]. The indirect chelation method utilizes a bifunctional chelator (Figure 1). Ogawa et al. demonstrated that indirect chelation eliminates the radionuclidic disassociation during bone binding [10]. In addition, encouraging advances were seen in the development of [^68^Ga]Ga-BPAMD, [^68^Ga]Ga-EDTMP, [^68^Ga]GaNO2A^BP^, [^68^Ga]Ga-DOTA^ZOL^, and [^68^Ga]Ga-DOTA^PAM^ [11,12,13]. A promising preclinical study for [^68^Ga]Ga-DOTA^ZOL^ had led to the preparation of radiolabeled [^177^Lu]Lu-DOTA^ZOL^ for therapeutic purposes. However, most of the findings utilize 1,4,7,10-tetraazacyclododecane-1,4,7,10-tetraacetic acid (DOTA) chelator. Hence, to diversify newer findings in this field, the present work deems to prepare and characterize conjugated NODAGA-pamidronic acid (NODPAM) precursor and optimize the radiolabeling of [^68^Ga]Ga-NODAGA-pamidronic acid ([^68^Ga]Ga-NODPAM) as a potential PET bone imaging agent. Finally, the potential uses of [^68^Ga]Ga-NODPAM will be determined via in vitro bone binding assay and in vivo animal biodistribution studies.

## 2. Experimental

### 2.1. Materials and Reagents

**Figure 2:**
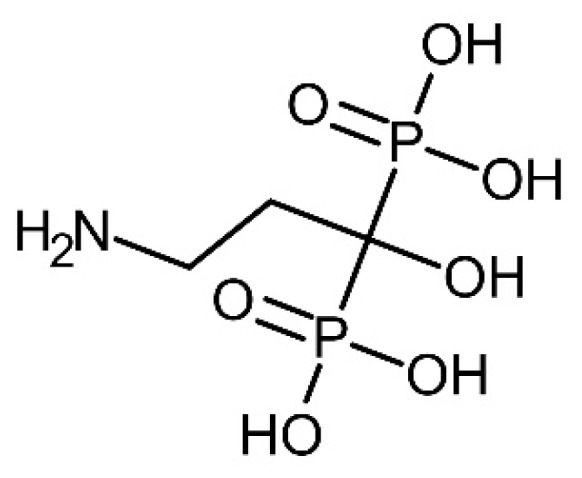
Chemical structure of pamidronic acid.

**Figure 3:**
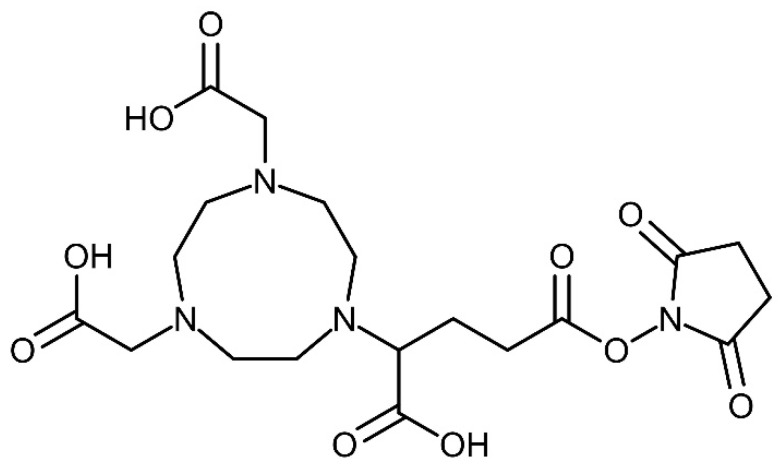
Chemical structure of NODAGA-NHS.

Pamidronic acid (chemical structure showed in Figure 2) was purchased from Santa Cruz Biotechnology Inc., Dallas, TX, USA. The 2,2-(7-(1-carboxy-4-((2,5-dioxopyrrolidin-1-yl)oxy)-4-oxobutyl)-1,4,7-triazonane-1,4-diyl)diacetic acid (NODAGA-NHS) (chemical structure showed in Figure 3) bifunctional chelator was obtained from CheMatech Dijon, France. Triethylamine (TEA) and dimethylformamide (DMF) were purchased from Sigma Aldrich, St. Louis, MO, USA, and Qrec, New Zeeland, respectively. The solid-phase extraction (SPE) C18 cartridge used was obtained from Waters, Milford, MA, USA. The Millex-GN syringe nylon filters (0.20 µm, 13 mm diameter) were purchased from Merck, Darmstadt, Germany. For the in vitro bone binding assay, the synthetic hydroxyapatite was purchased from Sigma Aldrich, St. Louis, MO, USA.

### 2.2. Preparation of NODPAM Precursor

**Figure 4:**
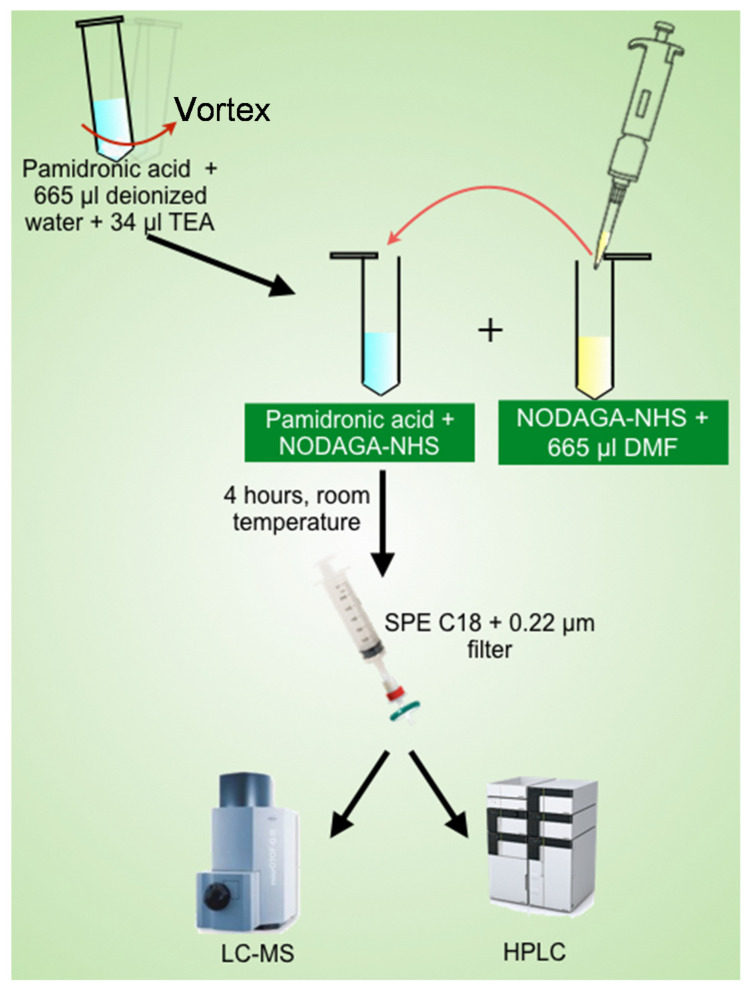
Schematic illustration of the NODPAM preparation process.

The preparation of NODPAM involves conjugation via the NHS ester strategy [14]. The pamidronic acid:NODAGA-NHS ratio was prepared according to Table 1. The weighed pamidronic acid was diluted with 665 µL of deionized water, and to it, 34 µL of TEA was added to adjust the pH to 8 and then vortexed until it dissolved entirely. Similarly, the NODAGA-NHS weighted was diluted with 665 µL of DMF, and from this, 665 µL of NODAGA-NHS were transferred in 50-µL fractions to the firstly prepared pamidronic acid solution. The reaction pH was monitored hourly using pH paper for 4 h in room temperature, and after that, the organic impurities trapped using an SPE C18 cartridge. The samples were filtered using a 0.20-µM nylon filter for liquid-chromatography mass spectrometry (LC-MS), reverse-phase high-performance liquid chromatography (RP-HPLC), and tandem mass spectrometry (MS^n^) analysis (Figure 4). The unreacted NODAGA chelator was removed using RP-HPLC and was freeze-dried accordingly.

### 2.3. RP-HPLC Method

The analytical RP-HPLC method was optimized using a reversed-phase C18 column (Shim-pack GIST 4.2 × 150 mm, 5 µm particle size, from Shimadzu, Kyoto, Japan) with 0.1% trifluoroacetic acid + water mobile phase adjusted to pH 2. The compounds were detected using a UV-VIS detector (SPD-20A, Shimadzu) at a wavelength of 220 nm, and the injection volume was 20 µL using a microlitre syringe (Glass capillary from Hamilton, Bonaduz, Switzerland). Three flow rates were used, 0.85, 0.65, and 0.50 mL/min. The retention time (RT) and the resolution were obtained from LC-Solution software. The peak identification of the unreacted NODAGA chelator was performed by determining the precision and repeatability injections of 1000 ppm NODAGA. The removal of the unreacted NODAGA chelator was performed using a fraction collector based on the optimized RP-HPLC method and unreacted NODAGA chelator peak identification.

### 2.4. Mass Spectrometry Analysis

The LC-MS analysis and tandem mass spectrometry (MS^n^) analysis were performed using different systems. The LC-MS spectra were obtained using an LC Dionex Ultimate 3000 (Thermo Scientific, Grand Island, NY, USA) equipped with an ESI-MS Bruker Daltonic MicroTOF Q detector. The chromatographic analysis was performed using a Gemini 5 μm NX-C18 110 Å 150 × 2 mm (Phenomenex, Torrance, CA, USA) column and an isocratic mobile phase of 0.1% formic acid + water. The flow rate and the temperature of the column throughout the analysis was 0.3 mL/min and 30 °C, respectively.

Tandem mass spectrometry was performed using a mass spectrometer equipped with electrospray ionization (ESI) and a triple Q-TOF analyzer (Triple TOF 5600, AB Sciex, Framingham, MA, USA). The chromatographic analysis condition was the same as the LC-MS analysis mentioned above except that it was performed on an Eksigent 110XL ekspert ultraLC System (AB Sciex, Framingham, MA, USA) using a Vydac Vision HT 5 μm C18 110 Å 100 × 2.1 mm (Grace, Leicestershire, UK) column. The ionization polarity set was in negative ion mode with the declustering potential at 80 V. The collision energy, collision spread, and nebulization energy was 40 eV, 1 eV, and 500 °C, respectively. The collision and nebulization gas were generated using purified nitrogen.

### 2.5. Radiolabeling of [^68^Ga]Ga-NODPAM

The ^68^Ga eluate was obtained from a ^68^Ge/^68^Ga Generator (iThemba LABS, Faure, South Africa; 30 mCi calibrated at 18-08-2016). Since this is an old generator, the highest concentration of ^68^Ga was eluted using 0.6 M hydrochloric acid by fractionally removing the first 0.5 mL and collecting the subsequent 2 mL of ^68^Ga eluate. The generator column was washed with another 2.5 mL of 0.6 M hydrochloric acid to ensure low metallic impurities in the following elutions. For the radiolabeling, approximately a 2.5 µg/50 µL (4 nmol) aliquot of NODPAM was reacted with 500 ± 10 µCi ^68^Ga. The reaction was buffered in 600 µL of 1.0 M NaOAc, and the pH was varied using different amounts of 1.0 M sodium hydroxide, as summarized in Table 2. The radiolabeling was also assessed at different temperatures (room temperature, 40, 60, and 80 °C) using a dry heating bath with a constant pH of 4.5. The radiochemical purity (RCP) was determined using a TLC-SG plate as a stationary phase and 0.4 M phosphate:acetonitrile (7:3) as a mobile phase and analyzed using a Radio TLC-Scanner (Scan-Ram, Lab Logic, Broomhill, Sheffield, UK).

### 2.6. In Vitro and In Vivo Assessment of [^68^Ga]Ga-NODPAM

The in vitro bone binding assay was performed according to the previous literature with slight modifications [12]. Approximately, 1.0 mL of normal saline was added to 20 mg of hydroxyapatite and refrigerated for 24 h. Precisely, 50 µL of radiolabeled [^68^Ga]Ga-NODPAM was pipetted into the prepared hydroxyapatite solution and was incubated for another 10 min at room temperature with moderate mixing on a vortex. The supernatant was carefully removed from the hydroxyapatite using a Pasteur pipette. The hydroxyapatite was then washed with 500 µL separating the [^68^Ga]Ga-NODPAM bound hydroxyapatite fraction from the unbound [^68^Ga]Ga-NODPAM. The percentage in vitro bone binding assay was determined by measuring both fractions using a gamma counter. The experiment was then repeated using [^99m^Tc]Tc-MDP and [^68^Ga]Ga-NODAGA as a comparison to [^68^Ga]Ga-NODPAM.

For the in vivo studies, two groups of 3 healthy male Sprague Dawley rats (*n* = 3) weighing 180–200 g were intravenously injected via the tail vein, under anesthesia using ketamine/xylazine, with 150 µCi (in 300 µL) [^68^Ga]Ga-NODPAM euthanized by cardiac exsanguination at 1 and 2 h post-injection (p.i). The organs of interest (liver, spleen, kidney, muscle, femur, lungs, heart, blood, stomach, and gut) were harvested, washed, and lastly, the uptake was measured using a gamma counter. The percentage injected dose per g (% ID/g) was calculated to determine the uptake in each organ. The % ID in the skeleton was calculated based on the skeleton weight of the rats (skeleton weight  =  9.66  +  0.0355 × bodyweight) [15]. The in vivo study was performed with approval from the Institutional Animal and Care Use Committee, Putra Malaysia University, authorization no. UPM/IACUC/AUP-R012/2019.

## 3. Results

### 3.1. LC-MS Chromatogram

**Figure 5:**
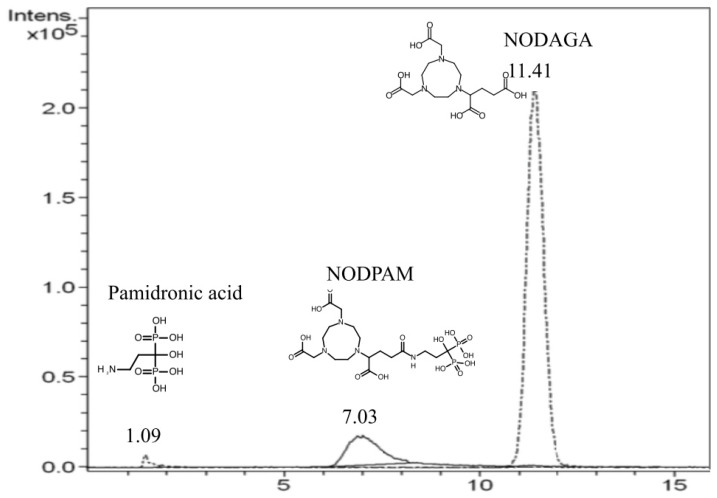
LC-MS chromatogram of unseparated NODPAM mixture (pamidronic acid:NODAGA (3:2)).

The liquid chromatographic condition was according to the ion-suppression reverse phase chromatography. Referring to Figure 5, the RT values for pamidronic acid (MW: 233.9 Da), NODPAM (MW: 591.1 Da), and unreacted NODAGA chelator (MW: 374.1 Da) were 1.09, 7.03, and 11.41 min respectively. The chromatogram shows that there is a difference in the polarity between these three compounds, with pamidronic acid being the most polar and the unreacted NODAGA chelator being the least polar. The % yield of NODPAM was estimated based on the ions detected relative to the unreacted NODAGA chelator. Referring to the result summarized in Table 3, the preparation yield of NODPAM was highest (39.91%) at a molar ratio of pamidronic acid:NODAGA-NHS of 19:2, followed by 9:2 (23.22%) and 3:2 (13.03%). This shows that there was a relation between the % NODPAM yield and the ratio of pamidronic acid:NODAGA-NHS, which supports Hermanson et al. for optimizing the product yield [16].

### 3.2. RP-HPLC Separation

**Figure 6:**
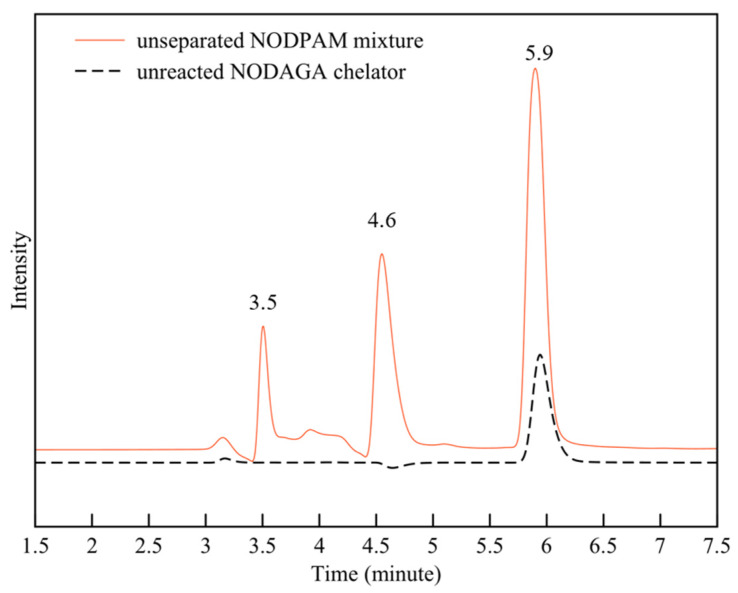
The identification of the unreacted NODAGA chelator RP-HPLC chromatogram from the unseparated NODPAM mixture (UV 220 nm, 0.1% TFA + water, flow rate: 0.5 mL/min).

**Figure 7:**
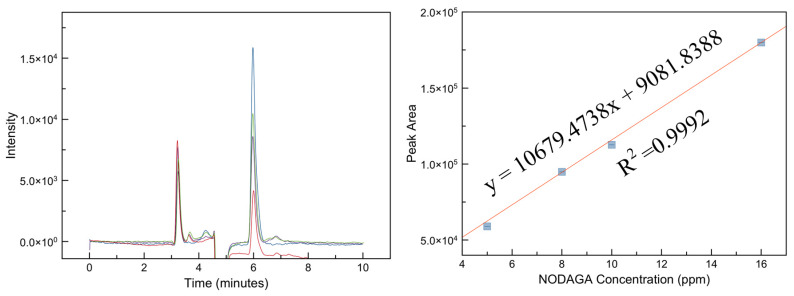
Linearity overlaid chromatograms and calibration curve for the unreacted NODAGA chelator; *R*^2^ = 0.9992, y = 10679.4738x + 9081.8388.

The chromatogram resolution defines the quality of separation between the peaks while the resolution is dependent on the RT among the two peaks and the baseline bandwidth, and so, to meet the system suitability test, the required resolution should be above 1.5 [17]. Based on the results obtained in Table 4, the resolution between the solvent peak and pamidronic acid was improved from 1.286 to 1.403 and 1.613 by decreasing the flow rate from 0.85 to 0.65 and 0.50 mL/min, respectively. Following the RP-HPLC optimized method, the pattern of the three peaks matched the LC-MS chromatogram (Figure 5). Consequently, the unreacted NODAGA chelator peak was identified at the RT of 5.9 min (Figure 6). The precision illustrated in Table 5 shows that the percentage relative standard deviation (%RSD) for the unreacted NODAGA chelator peak area and RT were 1.315% and 0.64%, respectively (less than 2%). The detection limit was calculated based on the linearity peak area (Figure 7; *R*^2^ = 0.9992, y = 10679.4738x + 9081.8388) against the NODAGA concentration (ppm), where we found the detection limit to be 50 ppb. Based on this result, the unreacted NODAGA chelator’s peak was removed using a fraction collector.

**Figure 8:**
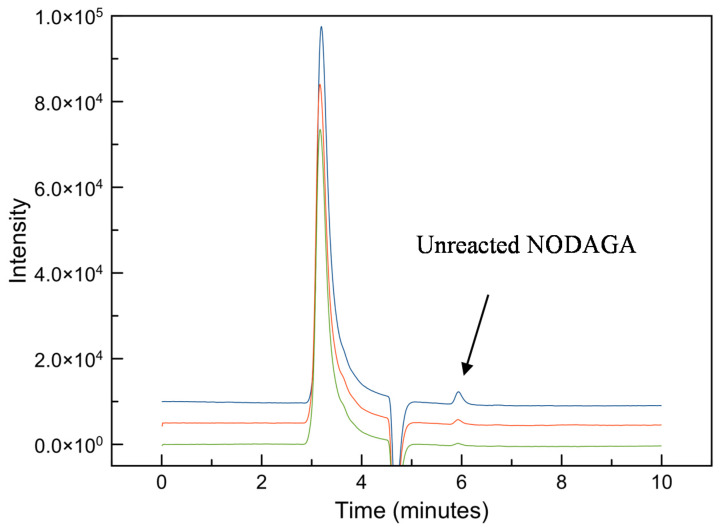
The re-analysis of the collected NODPAM precursor shows a considerable amount of unreacted NODAGA chelator.

**Figure 9:**
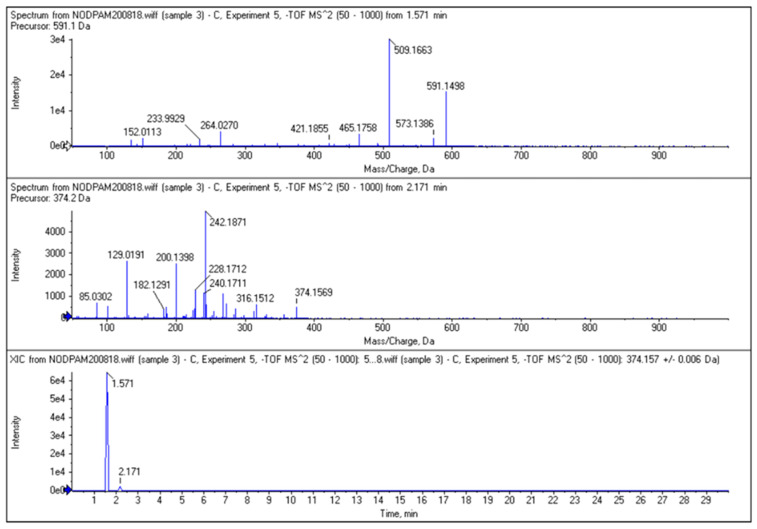
The MS^n^ and the chromatogram of the NODPAM ([M − H]^−^ *m*/*z* 591.15, RT = 1.571 min) precursor showing the presence of unreacted NODAGA chelator ([M − H]^−^ *m*/*z* 374.16, RT = 2.171 min) in the LC-MS^n^ analysis.

The re-analysis of the collected NODPAM precursor fraction shows a small amount of unreacted NODAGA chelator, as seen in the RP-HPLC (Figure 8) and LC-MS^n^. Based on the RP-HPLC result, the amount of unreacted NODAGA chelator present in the NODPAM precursor was approximately 0.68 ppm. This was also confirmed from the LC-MS^n^ analysis (Figure 9), whereby the NODPAM and unreacted NODAGA chelator elutes at RT 1.571 (MW: 591.1 Da) and 2.151 (MW: 374.2 Da), respectively. There were differences in the RT for the LC-MS^n^ analysis from the RP-HPLC analysis, which was likely due to differences in the column and mobile phase. 

### 3.3. Tandem Mass Spectrometry (MS^n^) Analysis

**Figure 10:**
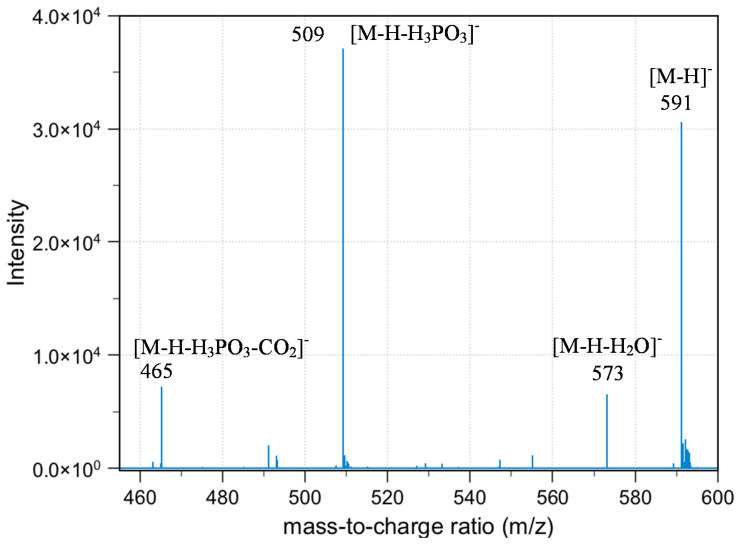
ESI-MS^n^ mass spectrum of NODPAM producing the 591 [M − H]^−^ precursor ion and 509 [M-H-H_3_PO_3_]^−^ base peak.

**Figure 11:**
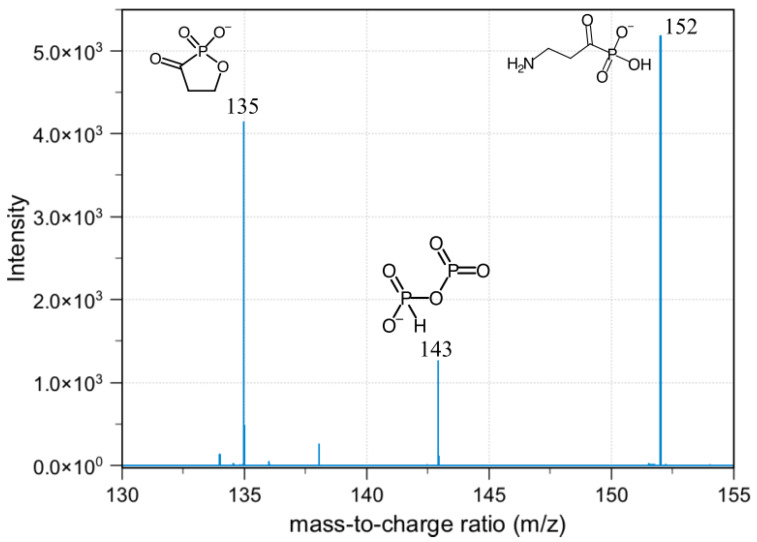
Diagnostic ion for bisphosphonates observed at *m*/*z* 143.

**Figure 12:**
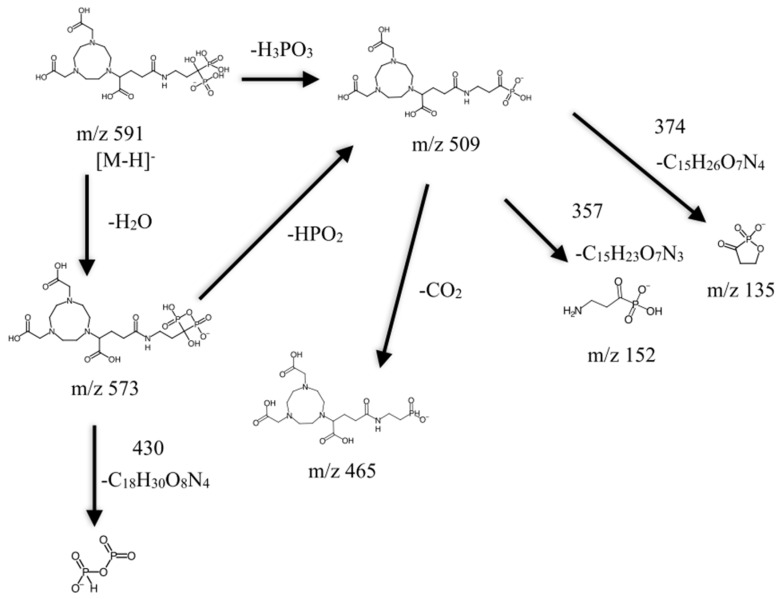
Proposed (−) ESI-MS^n^ fragmentation pathway for NODPAM.

As summarized in Figure 10, the neutral loss observed in this experiment was 18, 64, 82, and 44, which arises from the H_2_O, HPO_2_, H_3_PO_3_, and CO_2_, respectively. The ESI-MS^2^ produces two fragment ions, which were *m*/*z* 573 and 509, through the neutral loss of H_2_O and H_3_PO_3_, respectively. The *m*/*z* 573 was produced by -OH dehydration of two phosphorous groups forming a four-membered ring. The fragment ion for *m*/*z* 509 resembles [M-H-H_3_PO_3_]^−^. The ESI-MS^3^ from *m*/*z* 509 produces three fragment ions, which were observed at the *m*/*z* of 465, 152, and 132. The *m*/*z* 465 was produced from the neutral loss of carbon dioxide [M-H-H_3_PO_3_-CO_2_]^−^. The ESI-MS^3^ from *m*/*z* 573 produces a fragment ion of 143. The *m*/*z* 143 in the lower mass series was found to be an identical fragment ion for compounds containing a bisphosphonate group (Figure 11). Based on the fragments produced, the primary fragment ions were 573 [M-H-H_2_O]^−^, 509 [M-H-H_2_O-HPO_2_]^−^/[M-H-H_3_PO_3_]^−^, and 465 [M-H-H_2_O-HPO_2_-CO_2_]^−^/[M-H-H_3_PO_3_-CO_2_]^−^. The structures postulated in Figure 12 were based on the molecular formula, which abides the ring plus double bond equivalence (RDBE) and the nitrogen rule (N Rule) in Table 6 [18].

### 3.4. Radiolabeling Optimization

**Figure 13:**
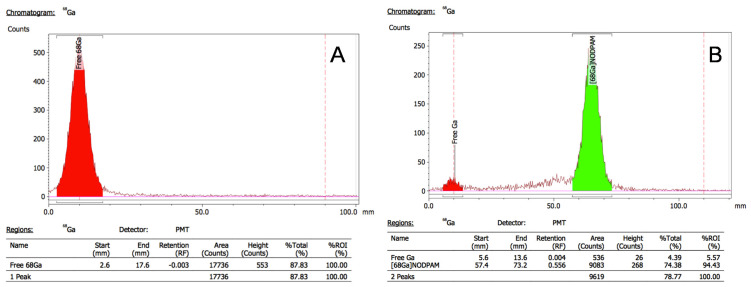
Radiochemical purity analysis for [^68^Ga]Ga-NODPAM using TLC-SG (silica gel) plate and 0.4 M phosphate:acetonitrile (7:3) as the mobile phase (**A**)—Free gallium-68; (**B**)—Radiolabeled [^68^Ga]Ga-NODPAM.

**Figure 14:**
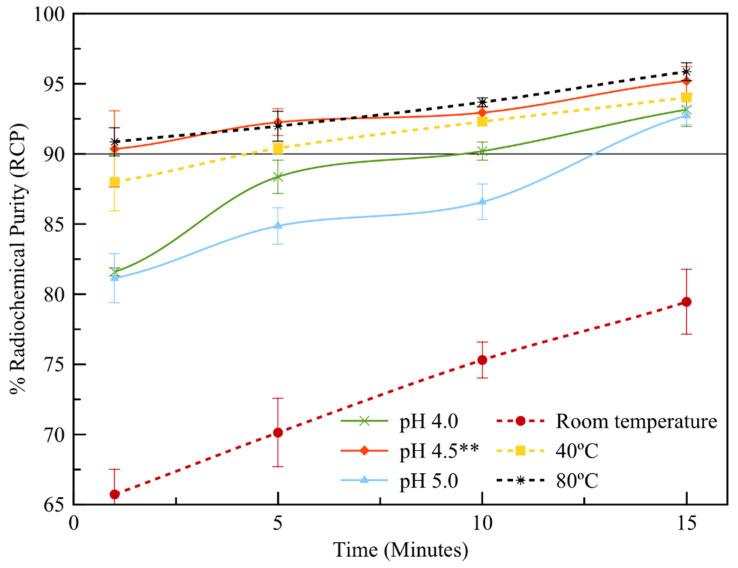
The optimization of %RCP at three different pH (constant temperature: 60 °C, NODPAM precursor: 4 nmol) and the temperature (constant pH 4.5, NODPAM precursor: 4 nmol) reacted in 500 µCi ± 10. ** reaction of 60 °C.

The radiolabeling of [^68^Ga]Ga-NODPAM was determined based on the developed rTLC (radio thin layer chromatography) method (Figure 13) (Rf: ^68^Ga(III) = 0 and [^68^Ga]Ga-NODPAM = 0.5). Three main variables that influence the formation of [^68^Ga]Ga-NODPAM complex were studied: pH, temperature, and time. Based on the results summarized in Figure 14, 90% RCP was achieved within the first 15 min at pH ranging from 4 to 5 with a heat applied above 60 °C. The rate of [^68^Ga]Ga-NODPAM complex formation was highest at pH 4.5 and lowest at pH 5.0. 

The influence of temperature (Figure 14) was found to be significant towards %RCP (*p* < 0.05) when comparing the reaction at room temperature and 40 °C. A higher rate of reaction was seen at the 60 °C temperature (10 min: 92.76% ± 0.36, 15 min: 95.20% ± 1.01) and above (refer to pH 4.5**). However, there was not much difference between the reaction rate at temperatures of 60 and 80 °C. Hence, it can be said that the rate of reaction time was most efficient (10 min 93.69% ± 0.31, 15 min: 95.86% ± 0.63) at a temperature above 60 °C and pH of 4.5.

### 3.5. In Vitro Bone Binding Assay and In Vivo Biodistribution of [^68^Ga]Ga-NODPAM

**Figure 15:**
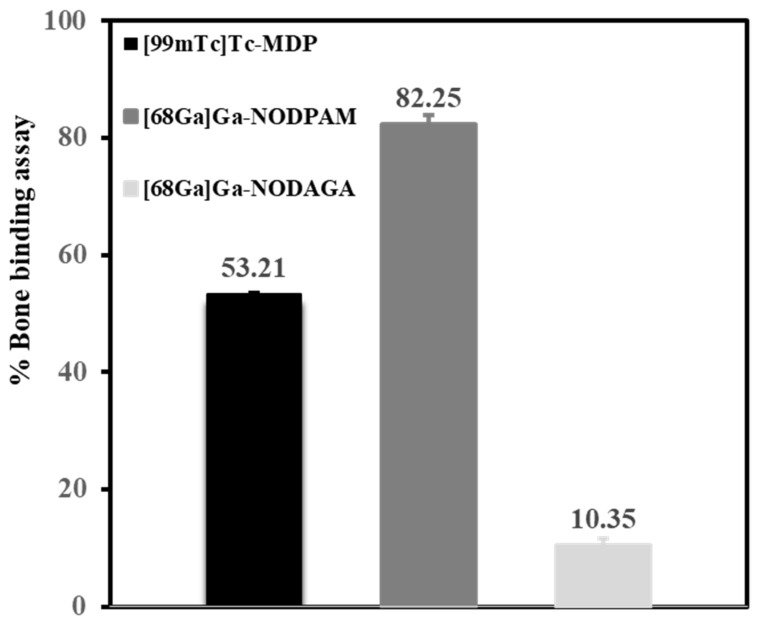
In vitro % bone binding assay for [^99m^Tc]Tc-MDP, [^68^Ga]Ga-NODPAM, and [^68^Ga]Ga-NODAGA.

The bone binding assay result illustrated in Figure 15 shows the bone binding capacity of [^68^Ga]Ga-NODPAM as compared to both the [^99m^Tc]Tc-MDP and [^68^Ga]Ga-NODAGA control. The [^68^Ga]Ga-NODAGA demonstrates almost no bone binding, with a percentage of 10.35% ± 1.29. The bone binding assay for [^68^Ga]Ga-NODPAM (82.25 ± 1.73%) was higher than [^99m^Tc]Tc-MDP (53.21 ± 0.28%), with a mean difference of 29.04% (*p* < 0.05). Hence, it can be said that the bone binding assay was better for [^68^Ga]Ga-NODPAM as compared to [^99m^Tc]MDP.

**Figure 16:**
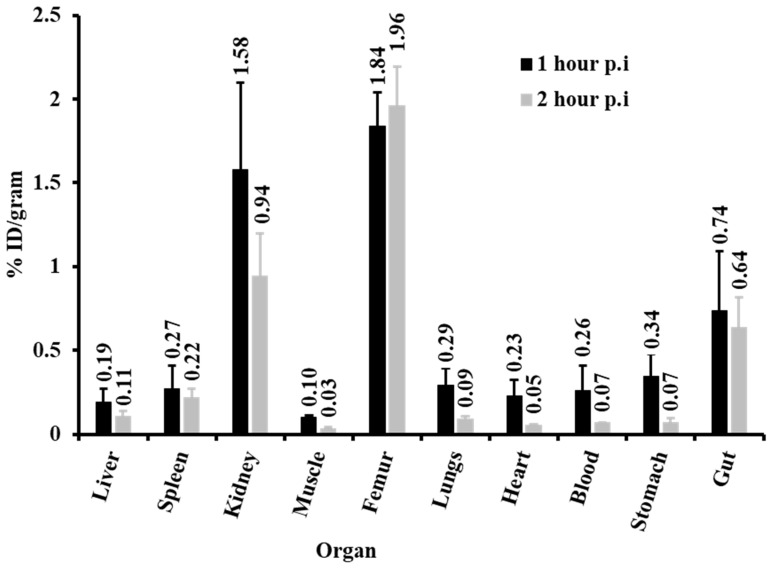
In vivo animal biodistribution of [^68^Ga]Ga-NODPAM in healthy Sprague Dawley rats examining its % ID/g of each organ of interest for 1 h and 2 h p.i. study.

The in vivo biodistribution of [^68^Ga]Ga-NODPAM in Figure 16 demonstrates an increase in % ID/g of [^68^Ga]Ga-NODPAM in the femur from 1.84% ± 0.20 (1 h p.i.) to 1.94% ± 0.23 (2 h p.i.). The kidney uptake was high, with % ID/g of 1.58% ± 0.52 and 0.94% ± 0.26 for 1 and 2 h p.i., respectively. The background uptake of 1 h p.i. for both blood and muscle was low, with % ID/g of 0.07% ± 0.01 and 0.03% ± 0.01, respectively. The calculated % ID in the skeleton depicted in Table 7 was found to be 31.25% ± 3.67 and 35.11% ± 3.02 for 2 h p.i. The bone-to-background ratio was highest at 2 h p.i. with the blood ratio of 27.53 and muscle ratio of 64.37.

## 4. Discussion

**Figure 17:**
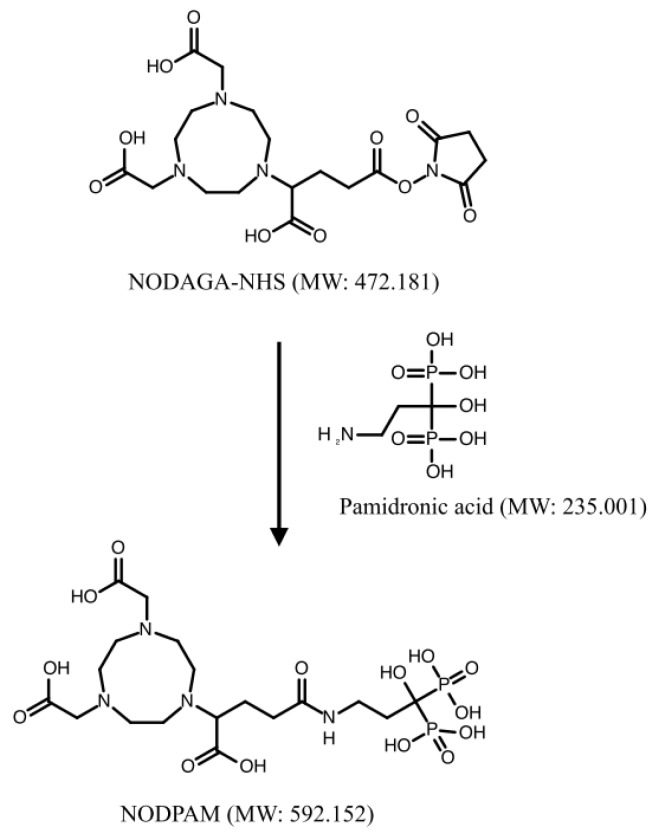
Conjugation of pamidronic acid and NODAGA-NHS producing NODPAM.

**Figure 18:**
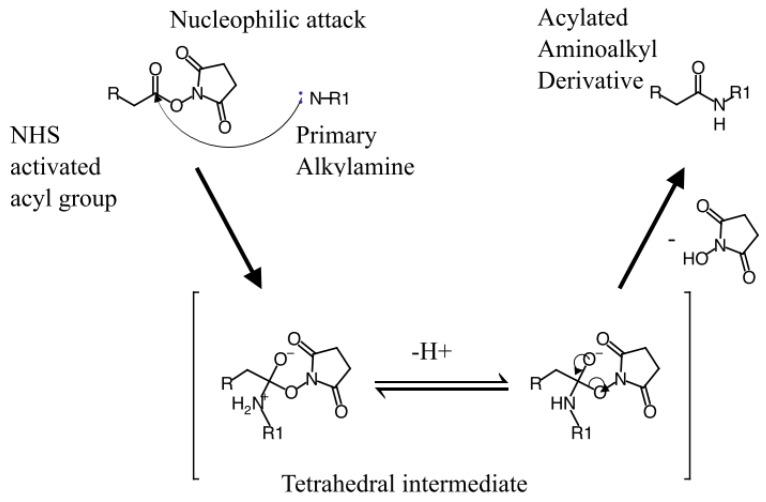
Acylation mechanism through the nucleophilic attack forming a tetrahedral intermediate and finally the product.

**Figure 19:**
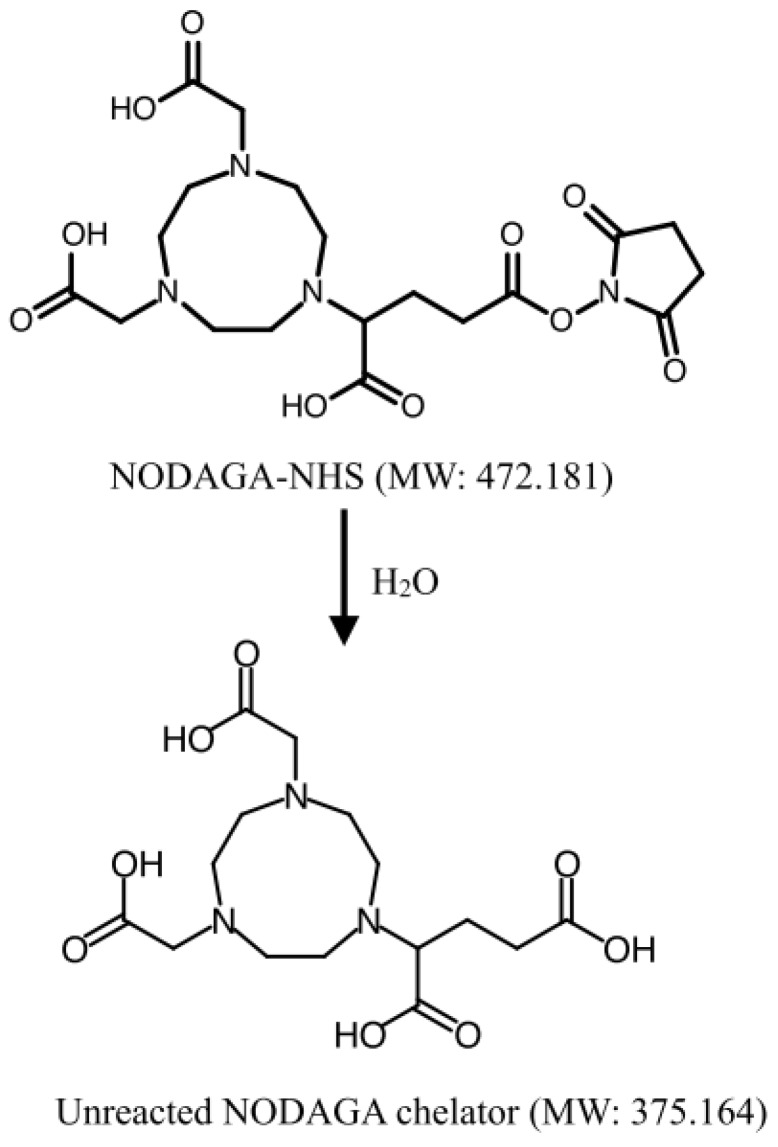
Hydrolysis of NODAGA-NHS into unreacted NODAGA chelator.

The preparation of NODPAM includes conjugation of the NODAGA chelator with pamidronic acid as the targeting molecule via the NHS ester strategy (Figure 17), where the reaction conditions include the pH of 8 at room temperature for 4 h [16]. The reaction mechanism includes the attacking of the primary amine onto the carbonyl group, followed by the formation of the tetrahedral intermediate, and finally, the removal of the leaving group occurs (Figure 18). The nucleophilicity of the primary amine is more potent as compared to the oxygen-containing group; R-NH_2_ > R-O^−^. However, the presence of water may have caused hydrolysis to the NODAGA-NHS. Hence, the unconjugated NODAGA-NHS would appear as hydrolyzed unreacted NODAGA chelator (Figure 19). Therefore, the detection of unreacted NODAGA chelator was at an exact molecular weight of 375.1 g/mol ([M − H]^−^ *m*/*z* = 374.1) in the mass spectrometry analysis.

The product yield was affected by the pamidronic acid:NODAGA-NHS ratio. An increase in the pamidronic acid molar ratio maximizes the modification of amines and reduces the effect of hydrolysis. Besides, the reaction was effective when the NODAGA-NHS was diluted in DMF instead of an aqueous solution. The higher pH may also increase the rate of reaction; however, this may compromise the yield due to hydrolysis [19,20,21]. Instead, the pH was regulated using an organic base, which, in this case, was TEA (good proton acceptor).

The ion-suppression reverse-phase LC-MS and RP-HPLC method were able to separate all the three polar compounds accordingly. Generally, reverse-phase columns are hydrophobic. However, the ion-suppression method enables better polar compound retention using the C18 column [22]. Thus, the retention is dependent on the pKa of each compound. In an acidic mobile phase, a lower pKa compound retains less as compared to the higher pKa compound. Hence, based on the chromatogram, it can be said that the degree of ion suppression increases accordingly from pamidronic acid, NODPAM to unreacted NODAGA chelator.

Nevertheless, a mute amount of unreacted NODAGA was seen in the collected NODPAM fraction, which was also confirmed with LC-MS analysis. The C18 column used in the RP-HPLC method might not be suitable for the ion-suppression method. Instead, the hydrophilic interaction chromatography (HILIC) type of column may improve the separation method. There was a shift in the RT for the RP-HPLC and LC-MS analysis results, and this was mainly because of the differences in the column size and the mobile phase used between the two systems. The mobile phase used in the RP-HPLC analysis was 0.1% trifluoroacetic acid + water while 0.1% formic acid + water was used for the LC-MS analysis. The rationale of these differences is that trifluoroacetic acid has a lower pKa value compared to formic acid, which warrants better retention and separation for RP-HPLC analysis. However, trifluoroacetic may not be suitable for LC-MS analysis due to its large molecular weight causing an interference. Thus, the use of a mobile phase with a smaller molecular weight solvent, such as formic acid, lowers the interference in LC-MS analysis, especially when analyzing small molecules.

**Figure 20:**
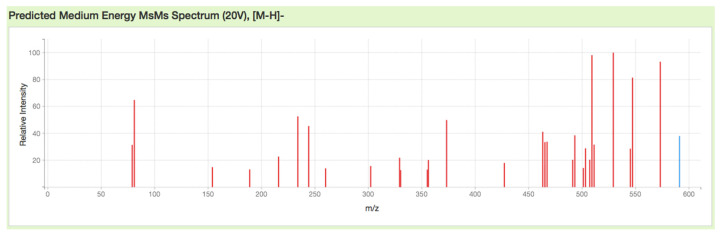
Predicted [M − H]^−^ fragment ions for NODPAM using competitive fragmentation modelling software.

Mass spectrometry plays an important role in the preparation of radiolabeled precursors, and so in this experiment, mass spectrometry analysis was fully utilized to characterize the NODPAM’s structure. From the MS^n^ analysis, the base peak was obtained at the *m*/*z* value of 509, corresponding to two possible pathways for the production of fragment ion *m*/*z* 509: [M-H-H_2_O-HPO_2_]^−^ and [M-H-H_3_PO_3_]^−^ [23]. The abundance of 509 fragment ion was due to the (1) stability and (2) low proton affinity of the neutral loss (Field’s rule). The increase in the unsaturation of *m*/*z* 509 based on the RDBE reflects increasing stability. Further fragmentation of the base peak 509 by CO_2_ neutral loss produces the 465 fragment ion. The *m*/*z* 573 and 509 fragment ions also produce characteristic ions at a lower mass series. In the lower mass series, the three fragment ions were observed at the *m*/*z* values of 152, 143, and 135. The fragment ions produced were based on the even-electron rule, which favors the heterolytic process through the charge–retention–fragmentation pathway [24]. The characteristic ion observed at *m*/*z* 143 depicts the presence of a bisphosphonate group [23]. The structures postulated were in agreement with other literature related to the bisphosphonates fragmentation pathway [25]. The fragment ions for NODPAM were also confirmed with the competitive fragmentation modelling software (Figure 20) [26], and we believe that this is a reliable technique to predict the outcome of an MS^n^ analysis. The free online software provides a simple import of structures in SMILES or InChI format for predicting the spectra. The technique is suited for beginners in using MS to forecast the fragmentation of their compound prior to the analysis.

One of the main issues in ^68^Ga radiolabeling is the formation of colloids, i.e., ^68^Ga element is susceptible to complex formation with OH^−^ at higher pH to form (^68^Ga)OH_3_ colloid, causing slow ligand interchange with the designated chelator. Failure to eliminate (^68^Ga)OH_3_ colloid formation alters the biodistribution, thereby causing accumulation in the liver [27,28]. However, a basic donor (NODPAM precursor) demands a slightly higher pH for the metal–chelate complex to be able to form. Hence, a balance in pH is vital to assure both a hard acid (^68^GaCl_3_) and strong Lewis base complex. Thus, in this research, ^68^Ga eluate was weakly complexed with acetate buffer before the reaction with NODPAM precursor to limit the event of [^68^Ga]Ga-OH_3_ colloid formation. In our experience, this method empirically protects ^68^Ga from forming colloid and increases the [^68^Ga]Ga-NODPAM radiolabeling efficiency [29].

By applying heat into the reaction, the complexation rate of [^68^Ga]Ga-NODPAM increases—the stable complex forms with sufficient activation energy transferred to the chelator and ^68^Ga. The activation energy required for a cyclic type of chelator, in general, was high as compared to an acyclic type of chelator, with the latter potentially compromising the stability. However, comparing two cyclic types of chelators, which are NODAGA and DOTA, the former requires lower activation energy as compared to the latter. For example, the use of a DOTA chelator for bone imaging applications, such as [^68^Ga]Ga-BPAMD, requires a temperature of 100 °C and reaction time of 20 min [30]. Inversely, the temperature needed for [^68^Ga]Ga-NODPAM complex formation was considerably milder, which was at 60 °C within 10 min. This relates to the NODAGA chelator’s cavity size, which accommodates ^68^Ga’s small ionic radius, consequently achieving better radiolabeling efficiency and in vivo stability [31,32,33]. Therefore, the radiolabeling efficiency of [^68^Ga]Ga-NODPAM was ideally improved as compared to any ^68^Ga-DOTA conjugates [34]. It is also important to note that NODAGA conjugate can radiolabel at room temperature within 5–10 min [35,36,37]. However, this warrants a greater amount of precursor and a more specific pH in order to achieve a higher yield.

There are two ascertained parameters in assessing the ^68^Ga’s radiopharmaceutical: (1) Target binding affinity, and (2) biodistribution. The former depicts how a radiopharmaceutical may attach or bind to a specific target, and at the same time, the latter is the foundation of a radiopharmaceutical reaching the specific target without imparting unnecessary radiation to the non-target organs [38]. In the case where ^68^Ga is used as a radionuclide, the radiopharmaceutical must be able to have fast target localization, good blood clearance, and high renal excretion primarily due to its short half-life [39]. In this work, the structure of the NODPAM precursor consists of pamidronic acid (targeting molecule) and NODAGA (bifunctional chelator). The structure of the precursor was based on two considerations: (1) Pamidronic acid’s bone binding assay, and (2) NODAGA bifunctional chelator’s radiolabeling efficiency. Referring to a study conducted by Ebitino et al., the bone binding assay for different bisphosphonates was pamidronic acid > alendronic acid > zoledronic acid > risedronic acid > ibandronic acid [40], conclusively showing the ability of pamidronic acid in binding highly osteoclastic active sites as compared to other bisphosphonates.

**Figure 21:**
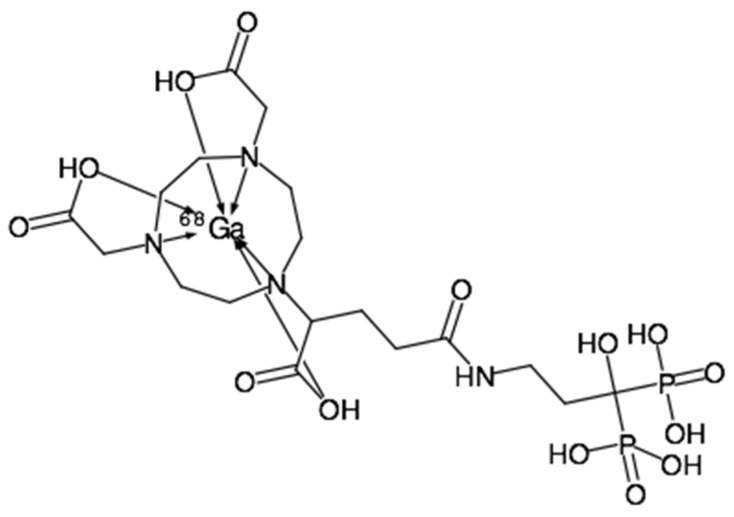
The structure of radiolabeled [^68^Ga]Ga-NODPAM.

The statement made was reflected in the in vitro bone binding assay and the in vivo animal biodistribution study. The [^68^Ga]Ga-NODPAM in vitro bone binding assay showed higher retention as compared to the conventional [^99m^Tc]Tc-MDP. The main difference between these two radiopharmaceuticals is that [^99m^Tc]Tc-MDP forms direct chelation between the radionuclide and the targeting molecule, which may disassociate during the bone binding process. Unlike [^99m^Tc]Tc-MDP, the phosphate groups in the [^68^Ga]Ga-NODPAM structure (Figure 21) did not interfere in ^68^Ga complexation. Thus, tethering the pamidronic acid with NODAGA eliminates the likelihood of [^68^Ga]Ga-NODPAM disassociation into ^68^Ga and NODPAM [10]. Besides, pamidronic acid forms two more bonds from the hydroxyl (α-OH) and amine groups, which will enhance the bone binding affinity [41]. 

The in vitro bone binding assay result was reproduced in the in vivo animal biodistribution studies. The [^68^Ga]Ga-NODPAM accumulates in the bones within 1 h p.i., with low uptake in both blood and muscle. Fast renal clearance was also observed in this experiment, which was contributed mainly by the ionic structure of [^68^Ga]Ga-NODPAM. High renal retention was seen, which might be due to residues of ^68^Ga-labeled unreacted NODAGA as shown in Figure 9. Despite the fact, the bone-to-blood ratio of 1 h p.i. was slightly lower than a previous study (7.6), which used DOTA as a chelator for pamidronic acid ([^68^Ga]Ga-DOTA^PAM^) [12]. Though the influence of NODAGA chelator was seen to improve the target affinity in other studies, it was not observed in this experiment [42]. One particular reason would be the highly ionic structure of both [^68^Ga]Ga-NODPAM and [^68^Ga]Ga-DOTA^PAM^, which may reduce the interaction time with the target, thus giving almost the same outcome. In comparison, Pfannkuchen et al. showed substantial improvements in the bone-to-blood ratio for [^68^Ga]Ga-NODAGA-zoledronic acid over [^68^Ga]Ga-DOTA-zoledronic acid [8]. This could be due to the presence of an aromatic ring, which temporarily increases the interaction time between the ^68^Ga-labeled bisphosphonate with the target. Thus, based on this experiment, the influence of NODAGA may not improve the overall bone-to-background ratio as compared to the DOTA chelator, specifically when conjugated to pamidronic acid [43].

Nevertheless, the mild radiolabeling condition for [^68^Ga]Ga-NODPAM may suit routine clinical practice in addition to less wastage in radioactivity due to the long preparation time. Besides, shorter preparation time warrants lower radiation exposure to radiopharmacists. Hence, as a whole, this may signify the potential use of [^68^Ga]Ga-NODPAM as a bone imaging radiopharmaceutical. Furthermore, the developments in PET imaging tools and technology may suitably one day substitute SPECT imaging ([^99m^Tc]Tc-MDP). Hence, further studies, especially in animal biodistribution and PET-CT imaging using pathologically induced animals, are required before moving into clinical studies.

## 5. Conclusions

In conclusion, we indicate here the potentials of [^68^Ga]Ga-NODPAM as a radiopharmaceutical for bone imaging applications where the complex was prepared, characterized, and radiolabeled accordingly. From the analysis, we found that the NODPAM precursor was successfully prepared via the NHS ester conjugation strategy. The LC-MS and RP-HPLC chromatogram showed a similar pattern in addition to the MS^n^ results, which confirmed the NODPAM’s structure. The fragments produced from the MS^n^ result were verified using computed fragmentation modelling software. The rate of reaction for radiolabeling of [^68^Ga]Ga-NODPAM was found to be highest at pH 4.5 and at a temperature of 60 °C and above. We believe that the results generated in this work may add to the development of other ^68^Ga-based PET for bone imaging contrasting agents. In the present experience, the use of NODAGA bifunctional chelators may shorten the preparation time besides improving the in vivo stability. Apart from the PET imaging, NODPAM is also readily labeled with other radionuclides, such as copper-64, indium-111, and gallium-67, as this may expand the use of NODPAM for therapeutic purposes. However, some specific preclinical studies have to be performed to realize the potentials of NODPAM as a bone radiopharmaceutical. This includes radiolabeled [^68^Ga]Ga-NODPAM in vitro plasma stability, more advanced in vivo animal biodistribution studies including more time points and pathologically induced animals, and finally, in vivo animal PET imaging. With regards to the previous survey [4], there are ample opportunities to perform a comparison study between [^68^Ga]Ga-NODPAM and [^99m^Tc]Tc-MDP for PET bone imaging in Malaysia.

## Figures and Tables

**Table 1:** Pamidronic acid and NODAGA-NHS molar ratio.

	Pamidronic Acid (mg)	NODAGA-NHS (mg)	Pamidronic Acid:NODAGA-NHS
1	14.8	30.4	3:2
2	15.1	10.1	9:2
3	16.1	5.2	19:2

**Table 2:** The amount of 1.0 M NaOH (µL) added for determining the radiolabeling effect on pH.

1.0 M NaOH (µL)	pH
70	4.0
170	4.5
270	5.0

**Table 3:** Molar ratio effect on % yield of NOPDAM based on the peak area of LC-MS chromatogram.

Pamidronic Acid:NODAGA Molar Ratio	Peak Area		% Yield
	NODPAM	Unreacted NODAGA	
3:2	963,778	6,434,123	13.03
9:2	5,678,911	18,781,313	23.22
19:2	5,462,887	8,226,266	39.91

**Table 4:** Resolution of the three peaks (pamidronic acid, NODPAM, unreacted NODAGA) from the unseparated NODPAM mixture at different flow rates.

Flow Rate (mL/min)	Resolution (USP)		
	Pamidronic Acid	NODPAM	Unreacted NODAGA
0.85	1.286	3.611	4.041
0.65	1.403	4.160	4.451
0.50	1.613	4.553	4.660

**Table 5:** The precision of the unreacted NODAGA chelator 1 mg/mL based on the peak area and retention time.

Replicate	Peak Area	RT
1	9,897,445	6.07
2	9,891,724	6.01
3	9,670,956	5.99
Mean	9,820,041.67	6.02
SD	129,143.66	0.04
%RSD	1.32	0.64

**Table 6:** The relative error (ppm) and RDBE for each fragment produced from the MS/MS analysis.

Obtained *m*/*z*	Exact *m*/*z*	Relative Error (ppm)	RDBE	Molecular Formula
591.1498	591.1468	5.0749	5.5	C_18_H_33_N_4_O_14_P_2_
573.1386	573.1363	4.0130	6.5	C_18_H_31_N_4_O_13_P_2_
509.1663	509.1649	2.7496	6.5	C_18_H_30_N_4_O_11_P_1_
465.1758	465.1750	1.7198	5.5	C_17_H_30_N_4_O_9_P_1_
152.0113	152.0112	0.6578	1.5	C_3_H_7_N_1_O_4_P_1_
142.9298	142.9299	0.6996	1.5	H_1_O_5_P_2_
134.9850	134.9847	2.2225	2.5	C_3_H_4_O_4_P_1_

**Table 7:** The % ID in skeleton and bone-to-blood/muscle ratio for [^68^Ga]Ga-NODPAM 1 h and 2 h p.i.

Time Point	% ID in Skeleton (SD)	Bone-to-Blood Ratio	Bone-to-Muscle Ratio
1 h p.i.	31.25 (3.67)	6.94	18.19
2 h p.i.	35.11 (3.02)	27.53	64.37
